# Effects of 2‐year calorie restriction on circulating levels of IGF‐1, IGF‐binding proteins and cortisol in nonobese men and women: a randomized clinical trial

**DOI:** 10.1111/acel.12400

**Published:** 2015-10-06

**Authors:** Luigi Fontana, Dennis T. Villareal, Sai K. Das, Steven R. Smith, Simin N. Meydani, Anastassios G. Pittas, Samuel Klein, Manjushri Bhapkar, James Rochon, Eric Ravussin, John O. Holloszy, the CALERIE Study Group

**Affiliations:** ^1^ Department of MedicineWashington University School of MedicineSt LouisMOUSA; ^2^ Department of Clinical and Experimental SciencesUniversity of Brescia Medical SchoolBresciaItaly; ^3^ CEINGE Biotecnologie AvanzateNapoliItaly; ^4^ Center for Translational Research on Inflammatory Diseases (CTRID)Baylor College of MedicineMichael E DeBakey VA Medical CenterHoustonTXUSA; ^5^ Jean Mayer USDA Human Nutrition Research Center on AgingTufts UniversityBostonMAUSA; ^6^ Pennington Biomedical Research CenterBaton RougeLAUSA; ^7^ Translational Research Institute for Metabolism and DiabetesFlorida HospitalSanford Burnham Medical Research InstituteOrlandoFLUSA; ^8^ Duke Clinical Research InstituteDurhamNCUSA; ^9^ Rho Federal SystemsChapel HillNCUSA

**Keywords:** calorie restriction, cancer, cortisol, IGF‐1, IGFBP‐1, weight loss

## Abstract

Young‐onset calorie restriction (CR) in rodents decreases serum IGF‐1 concentration and increases serum corticosterone levels, which have been hypothesized to play major roles in mediating its anticancer and anti‐aging effects. However, little is known on the effects of CR on the IGF‐1 system and cortisol in humans. To test the sustained effects of CR on these key hormonal adaptations, we performed a multicenter randomized trial of a 2‐year 25% CR intervention in 218 nonobese (body mass index between 22 and 27.8 kg m^−2^) young and middle‐aged (20–50 years age range) men and women. Average CR during the first 6 months was 19.5 ± 0.8% and 9.1 ± 0.7% over the next 18 months of the study. Weight loss averaged 7.6 ± 0.3 kg over the 2‐years period of which 71% was fat mass loss (*P* < 0.0001). Average CR during the CR caused a significant 21% increase in serum IGFBP‐1 and a 42% reduction in IGF‐1:IGFBP‐1 ratio at 2 years (*P* < 0.008), but did not change IGF‐1 and IGF‐1:IGFBP‐3 ratio levels. Serum cortisol concentrations were slightly but significantly increased by CR at 1 year only (*P* = 0.003). Calorie restriction had no effect on serum concentrations of PDGF‐AB and TGFβ‐1. We conclude, on the basis of the present and previous findings, that, in contrast to rodents, humans do not respond to CR with a decrease in serum IGF‐1 concentration or with a sustained and biological relevant increase in serum cortisol. However, long‐term CR in humans significantly and persistently increases serum IGFBP‐1 concentration.

## Introduction

Data from experimental and epidemiological studies indicate that insulin‐like growth factor (IGF)‐1 and its binding proteins play a role in the biology of aging and in the pathogenesis of several common cancers (Yu & Rohan, 2000; Renehan *et al*., 2004; Yakar *et al*., 2005; Fontana *et al*., 2010; Kopchick *et al*., 2014). Patients with acromegaly, who have high growth hormone and IGF‐1 levels, experience a 2‐fold increased risk of gastrointestinal cancers (Renehan *et al*., 2003), whereas patients with congenital deficiencies in IGF‐1 seem to be protected against the development of cancer (Shevah & Laron, 2007; Guevara‐Aguirre *et al*., 2011). Data from several genetic animal models of longevity have shown that reduced function mutations in the IGF‐1 signaling pathway have low circulating IGF‐1 levels, reduced cancer incidence, and increased maximal lifespan (Fontana *et al*., 2010). Moreover, in a study of 31 genetically diverse inbred mouse strains, the median lifespan was inversely correlated with plasma IGF‐1 levels (Yuan *et al*., 2009). IGF‐1 is a potent mitogenic growth factor, which promotes cell proliferation and differentiation, and inhibits apoptosis (Yakar *et al*., 2005). The inhibition of the IGF‐1 pathway causes several cellular and metabolic adaptations, including downregulation of growth pathways, upregulation of autophagic and apoptotic pathways, increased resistance to multiple toxic agents, and increased genome stability (Fontana *et al*., 2010; Bartke *et al*., 2013).

Calorie restriction (CR) without malnutrition is one of the most powerful interventions to slow aging and prevent cancer in laboratory strains of rodents (Albanes, 1987; Fontana *et al*., 2010; Longo & Fontana, 2010). Adult‐onset moderate CR also reduces cancer incidence by more than 50% in Rhesus monkeys (Colman *et al*., 2009; Mattison *et al*., 2012). It has been hypothesized that the powerful inhibitory effect of CR on spontaneous, chemically induced and radiation‐induced tumors can be mediated, at least in part, by a reduction in IGF‐1 levels and an increase in corticosteroid levels (Longo & Fontana, 2010). In rodents, CR decreases serum insulin‐like growth factor‐1 (IGF‐1) concentration by 20–40% and increases serum corticosterone levels by 30–50% (Breese *et al*., 1991; Sabatino *et al*., 1991; Dunn *et al*., 1997). Both adrenalectomy and IGF‐I supplementation abrogate the protective effect of CR on neoplastic progression (Pashko & Schwartz, 1992, 1996; Dunn *et al*., 1997; Stewart *et al*., 2005). Data from two small short‐term (6 and 12 months) randomized clinical trials and a cross‐sectional observational study have shown that CR does not increase serum cortisol or reduce serum IGF‐1 and IGF‐1:IGFBP‐3 ratio levels, unless protein intake is also reduced (Weiss *et al*., 2006; Fontana *et al*., 2008; Redman *et al*., 2010; Tam *et al*., 2014). However, there are currently no randomized controlled trials (RCT) in young–middle age lean or slightly overweight men and women, evaluating the long‐term effects of CR on serum IGF‐1, IGFBPs, and cortisol concentrations.

One of the purposes of this 2‐year multicenter randomized controlled trial (CALERIE) was to evaluate the effects of a 25% reduction in energy intake in a large number of nonobese young and middle‐aged men and women on the IGF‐1 axis, and other growth and hormonal factors modulated by CR, which have been implicated in the biology of aging and in the pathophysiology of cancer, such as PDGF‐AB, TGF‐β‐1, and cortisol.

## Results

**Figure 1:**
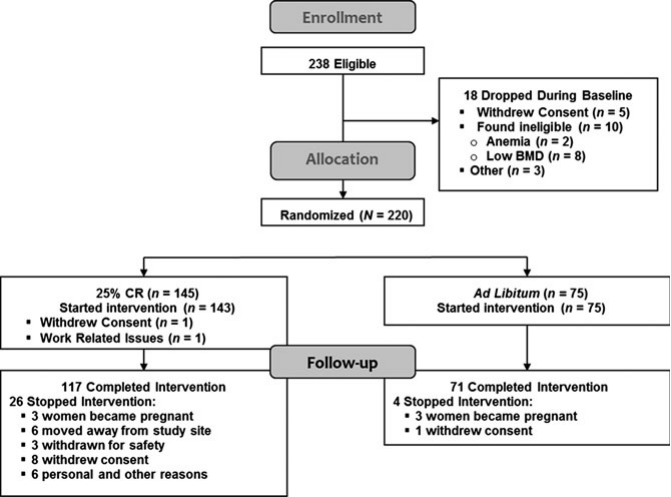
CONSORT diagram.

Of the more than 10 000 men and women assessed for eligibility, the screening procedures excluded 45% of them for their age or body mass index (BMI), 14% for health or medication reasons, and 30% refused to participate due to concerns about their ability to adhere to the protocol, and personal or study‐related issues (Ravussin *et al*., 2015). Of the 238 participants who began baseline assessments, 220 were randomized, 218 started the assigned intervention, and 82% of CR and 95% of ad libitum (AL), respectively, completed the study (Fig. 1). Table 1 presents sex, age, race, and BMI data for the two groups. Analysis revealed no significant differences between groups at baseline for these variables.

**Table 1:** Demographic, anthropometric, and clinical characteristics at baseline for the 218 participants who started the 2‐year intervention

	*Ad libitum* (*n* = 75)	Calorie restriction (*n* = 143)
Race		
White	57 (76%)	111 (77.6%)
African American	11 (14.7%)	15 (10.5%)
Other	7 (9.3%)	17 (11.9%)
Age (years)	37.9 (6.94)	38.0 (7.34)
Height (m)	168.4 (8.31)	168.9 (8.60)
Baseline weight (kg)	71.5 (8.65)	72.0 (9.49)
Baseline BMI (kg m^−2^)	25.1 (1.64)	25.2 (1.78)
Body fat (%)	33.6 (6.57)	32.9 (6.07)
FFM (kg)	47.6 (8.61)	48.5 (9.21)
Energy and macronutrient intake		
Energy intake (kcal day^−1^)	2045.3 (480.66)	2126.3 (558.60)
Protein (g kg^−1^ day^−1^)	1.2 (0.04)	1.2 (0.02)
Protein, % of energy	17.2 (3.48)	16.6 (3.04)
Fat, % of energy	34.7 (5.12)	33.5 (4.93)
Carbohydrates, % of energy	45.1 (6.33)	46.8 (6.48)
Laboratory values		
IGF‐1 (ng mL^−1^)	183.1 (49.40)	175.5 (42.66)
IGFBP‐1 (pg mL^−1^)	4477 (5033.7)	5459 (6252.0)
IGFBP‐3 (pg mL^−1^)	2529 (502.7)	2459 (399.8)
IGF‐1/IGFBP‐3 ratio	0.07 (0.025)	0.07 (0.020)
IGF‐1/IGFBP‐1 ratio	0.10 (0.105)	0.08 (0.092)
PDGF‐AB (ng mL^−1^)	20.0 (6.01)	18.1 (6.97)
TGF‐β1 (ng mL^−1^)	30.6 (8.02)	28.9 (9.72)
Cortisol (μg dL^−1^)	11.3 (5.91)	11.2 (4.92)

AL, *ad libitum* control group; CR, 25% calorie restriction group; FFM, fat‐free mass; FM, fat mass.

Values represent means (SD).

### Intervention adherence, body weight, and composition

Detailed information regarding the observed adherence to the intervention has been published previously (Ravussin *et al*., 2015). At baseline, there was no difference in average energy intake (assessed as TDEE during weight stability) between the CR [2467 (34) kcal day^−1^] and AL [2390 (45) kcal day^−1^] groups (*P* = 0.15). In the CR group, energy intake was reduced by 19.5 (0.8)% (480 kcal day^−1^) during the first 6 months, and by an average of 9.1 (0.7)% (234 kcal day^−1^) below baseline for the remaining 18 months of the study (*P* < 0.0001 vs. AL control group), while in the control group average daily energy intake was unchanged (Table 2). Weight loss averaged 8.3 (0.3) kg (11.5%) at 1 year and 7.6 (0.3) kg (10.4%) at 2 years in the CR group (*P* < 0.001), but did not change significantly in the AL group (Table 2). Body fat measured by dual‐energy X‐ray absorptiometry (DXA) decreased by 6.1 (0.2) kg at 1 year and 5.3 (0.3) kg at 2 years in the CR group (*P* < 0.001), but did not change in the AL group. Fat loss accounted for ~71% of the weight loss (Table 2).

**Table 2:** Change from baseline in body composition at 12 and 24 months in AL and calorie restriction (CR) groups

Outcome	AL		CR		Between‐group* P*‐value^b^
	Mean (SE)^a^	Within‐group* P*‐value^b^	Mean (SE)^a^	Within‐group* P*‐value^b^	
Clinical weight (kg)					
Baseline	71.5 (1.0)		72.0 (0.8)		0.978
Δ Month 12	−0.7 (0.4)	0.105	−8.4 (0.3)	< 0.001	< 0.001
Δ Month 24	0.1 (0.5)	1.0	−7.5 (0.4)	< 0.001	< 0.001
Body mass index (kg m^−2^)					
Baseline	25.1 (0.2)		25.2 (0.2)		0.937
Δ Month 12	−0.2 (0.1)	0.207	−2.9 (0.1)	< 0.001	< 0.001
Δ Month 24	0.1 (0.2)	1.0	−2.6 (0.1)	< 0.001	< 0.001
% Body fat					
Baseline	33.6 (0.8)		32.9 (0.5)		0.336
Δ Month 12	−0.47 (0.3)	0.254	−5.5 (0.2)	< 0.001	< 0.001
Δ Month 24	0.13 (0.3)	1.0	−4.6 (0.3)	< 0.001	< 0.001
Fat mass (kg)					
Baseline	23.8 (0.6)		23.5 (0.4)		0.611
Δ Month 12	−0.34 (0.3)	0.518	−6.1 (0.2)	< 0.001	< 0.001
Δ Month 24	0.38 (0.4)	0.564	−5.3 (0.3)	< 0.001	< 0.001
Fat‐free mass (kg)					
Baseline	47.6 (1.0)		48.5 (0.8)		0.475
Δ Month 12	−0.3 (0.2)	0.131	−2.2 (0.1)	< 0.001	< 0.001
Δ Month 24	−0.2 (0.2)	0.837	−2.2 (0.2)	< 0.001	< 0.001

a Baseline values are the observed mean (SE); change scores are the least‐squares adjusted means (SE) from the ITT repeated measures analysis.

b Within‐group *P*‐value tests for a significant change from baseline to the follow‐up time point in that group; between‐group *P*‐value tests for a significant between‐group difference in the change score at the time point. All *P*‐values reflect Bonferroni corrections, truncated at 1.0, as appropriate (see text).

### Self‐reported energy and nutrient intake

Seven‐day food records showed that the CR group significantly restricted their energy intake [−279 (29) kcal day^−1^ at 1 year and −216 (33) kcal d^−1^ at 2 years], while the AL [−83 (38) kcal day^−1^ at 1 year and −121 (43) kcal day^−1^ at 2 years] group maintained its intake (Table 3). Accordingly, intake of macronutrients such as fat also decreased in the CR compared with the AL group, but protein intake increased significantly (Table 3).

**Table 3:** Change from baseline in dietary energy intake and macronutrient composition at 12 and 24 months in AL and calorie restriction (CR) groups

Outcome	AL		CR		Between‐group* P*‐value^b^
	Mean (SE)^a^	Within‐group* P*‐value^b^	Mean (SE)^a^	Within‐group* P*‐value^b^	
Energy intake (kcal day^−1^)					
Baseline	2045.3 (55.5)		2126.3 (46.7)		0.463
Δ Month 12	−83.0 (38.6)	0.065	−279.5 (29.3)	< 0.001	< 0.001
Δ Month 24	−121.1 (42.7)	0.010	−216.3 (33.1)	< 0.001	0.073
Protein intake (g kg^−1^)					
Baseline	1.2 (0.04)		1.2 (0.02)		0.951
Δ Month 12	−0.002 (0.03)	1.0	0.11 (0.03)	< 0.001	0.007
Δ Month 24	−0.09 (0.04)	0.055	0.08 (0.03)	0.012	0.001
% Calories from protein					
Baseline	17.2 (0.4)		16.6 (0.3)		0.252
Δ Month 12	0.8 (0.4)	0.123	1.8 (0.3)	< 0.001	0.047
Δ Month 24	0.1 (0.4)	1.0	1.1 (0.3)	0.003	0.068
% Calories from fat					
Baseline	34.7 (0.6)		33.5 (0.4)		0.034
Δ Month 12	0.2 (0.6)	1.0	−4.8 (0.4)	< 0.001	< 0.001
Δ Month 24	0.5 (0.6)	0.827	−3.4 (0.5)	< 0.001	< 0.001
% Calories from carbohydrates					
Baseline	45.1 (0.7)		46.8 (0.5)		0.078
Δ Month 12	−0.7 (0.7)	0.578	3.6 (0.5)	< 0.001	< 0.001
Δ Month 24	−0.8 (0.7)	0.482	2.4 (0.5)	< 0.001	< 0.001

a Baseline values are the observed mean (SE); change scores are the least‐squares adjusted means (SE) from the ITT repeated measures analysis.

b Within‐group *P*‐value tests for a significant change from baseline to the follow‐up time point in that group; between‐group *P*‐value tests for a significant between‐group difference in the change score at the time point. All *P*‐values reflect Bonferroni corrections, truncated at 1.0, as appropriate (see text).

### IGF‐1 axis and other growth factors

The substantial and sustained increase in serum IGFBP‐1 concentration and the reduction in IGF‐1:IGFBP‐1 ratio in CR significantly exceeded changes in AL (Table 4). Serum IGFBP‐3 reductions in CR significantly exceeded those in AL at 12 months (*P* = 0.018), but not at 24 months (Table 4). In contrast, serum IGF‐1, IGF‐1:IGFBP‐3 ratio and PDGF‐AB levels did not change significantly between groups at 12 and 24 months (Table 4). Serum TGF‐β1 decreased from baseline at 12 and 24 months in CR (Table 4), but the declines did not differ significantly from the change in AL. Serum cortisol concentration slightly but significantly increased in the CR group at 12 months (*P* = 0.003), but not at 24 months (Table 4).

**Table 4:** Change from baseline in plasma concentrations of growth factors and cortisol at 12 and 24 months in AL and calorie restriction (CR) groups

Outcome	AL		CR		Between‐group* P*‐value^b^
	Mean (SE)^a^	Within‐group* P*‐value^b^	Mean (SE)^a^	Within‐group* P*‐value^b^	
IGF‐1 (ng mL^−1^)					
Baseline	183.1 (5.7)		175.5 (3.6)		0.589
Δ Month 12	−19.6 (4.9)	< 0.001	−7.1 (3.7)	0.108	0.072
Δ Month 24	−18.7 (4.1)	< 0.001	−15.1 (3.2)	< 0.001	0.919
IGFBP‐1 (pg mL^−1^)					
Baseline	4477 (585)		5459 (523)		0.088
Δ Month 12	409 (636)	1.0	1839 (474)	< 0.001	0.065
Δ Month 24	−616 (573)	0.568	1391 (443)	0.004	0.005
IGFBP‐3 (ng mL^−1^)					
Baseline	2528 (58.4)		2459 (33)		0.338
Δ Month 12	1 (43)	1.0	124 (32)	< 0.001	0.018
Δ Month 24	56 (49)	0.510	123 (38)	0.003	0.273
IGF‐1/IGFBP‐3 ratio					
Baseline	0.10 (0.00)		0.10 (0.00)		0.942
Δ Month 12	−0.008 (0.002)	< 0.001	−0.006 (0.002)	0.001	0.880
Δ Month 24	−0.008 (0.002)	< 0.001	−0.009 (0.002)	< 0.001	1.0
IGF‐1/IGFBP‐1 ratio					
Baseline	0.102 (0.012)		0.078 (0.008)		0.064
Δ Month 12	−0.007 (0.018)	1.0	−0.046 (0.014)	0.002	0.088
Δ Month 24	−0.020 (0.008)	0.018	−0.045 (0.006)	< 0.001	0.008
Cortisol (μg dL^−1^)					
Baseline	11.3 (0.69)		11.2 (0.41)		0.667
Δ Month 12	−0.91 (0.46)	0.102	0.78 (0.35)	0.055	0.003
Δ Month 24	−1.78 (0.51)	0.001	−0.44 (0.39)	0.530	0.312
PDGF‐AB (pg mL^−1^)					
Baseline	20 000 (699)		18 131 (583)		0.018
Month 12	−398 (628)	1.0	−26 (469)	1.0	1.0
Month 24	−681 (515)	0.375	−1465 (398)	< 0.001	0.426
TGF‐β1 (pg mL^−1^)					
Baseline	30 604 (932)		28 871 (813)		0.065
Δ Month 12	−3169 (932)	0.002	−3521 (697)	< 0.001	1.0
Δ Month 24	−5455 (707)	< 0.001	−6616 (549)	< 0.001	0.356

a Baseline values are the observed mean (SE); change scores are the least‐squares adjusted means (SE) from the ITT repeated measures analysis.

b Within‐group *P*‐value tests for a significant change from baseline to the follow‐up time point in that group; between‐group *P*‐value tests for a significant between‐group difference in the change score at the time point. All *P*‐values reflect Bonferroni corrections, truncated at 1.0, as appropriate (see text).

## Discussion

Research on aging and cancer mechanisms has shown that the IGF pathway is deeply implicated in the biology of aging and in pathogenesis of several common malignant and potentially deadly tumors (e.g., colon, prostate, breast and ovarian cancer) (3) and that diet (e.g., CR) influences cancer risk through this pathway (Renehan *et al*., 2004; Fontana *et al*., 2010; Longo & Fontana, 2010; Guevara‐Aguirre *et al*., 2011). Most research data in this field have been derived from animal, epidemiological and observational studies, because it is difficult and expensive to conduct long‐term randomized clinical trials in humans. Thus, this is the first adequately powered randomized clinical trial to test in nonobese humans the long‐term effects of CR on the IGF‐1 axis and other growth factors implicated in the pathogenesis of cancer.

The findings of this clinical trial showed that, unlike in rodents (Breese *et al*., 1991), long‐term CR results in a significant and persistent increase in serum IGFBP‐1 and decrease in IGF‐1:IGFBP‐1 ratio levels, which should translate into lower circulating levels of free IGF‐1 and inhibition of IGF‐1 activity in humans. IGFBP‐1 concentrations were 25.2% and 21% higher after 1 and 2 years of CR, respectively, resulting in a 42% lower IGF‐1:IGFBP‐1 ratio level in the CR group at 2 years. This increase in serum IGFBP‐1 levels in our CR research volunteers may be mediated by improved insulin sensitivity (Suikkari *et al*., 1988). In fact, serum concentration of IGFBP‐1, unlike IGFBP‐3 which binds 75–90% of circulating IGF‐I, is heavily influenced by the metabolic (i.e., insulin resistance, and insulin and glucagon levels) and nutritional (fasting and refeeding) state of the individual. Excessive adiposity‐induced insulin resistance and compensatory hyperinsulinemia have been shown to decrease hepatic synthesis of IGFBP‐1, which translates into increased concentrations of bioavailable IGF‐1, without modifications in serum total IGF‐1 levels (Lukanova *et al*., 2001; Maddux *et al*., 2006). Patients with type 1 diabetes have higher serum IGFBP‐1 concentrations than normoglycemic controls (Suikkari *et al*., 1988), and acute steady state hyperinsulinemia lowers serum IGFBP‐1 levels by 40–70% in normal individuals (Yeoh & Baxter, 1988; Snyder & Clemmons, 1990). Moreover, it has been shown that circulating levels of IGFBP‐1 are acutely increased by 3–4 fold in response to overnight fasting and decline rapidly after a meal (Busby *et al*., 1988; Smith *et al*., 1995).

Fasting, but not long‐term CR, lowers serum IGF‐1 concentration in humans into the range observed for growth hormone‐deficient patients (Thissen *et al*., 1994; Fontana *et al*., 2008; Redman *et al*., 2010). Consistent with these previous human studies, we found that, unlike in rodents, the reduction in energy intake in the CR group was not accompanied by a decrease in serum IGF‐1 concentration or IGF‐1/IGFBP‐3 ratio, even in younger men and women. One possible explanation for the lack of effect of CR on IGF‐1 levels in this and other studies may be the chronic high‐normal intake of dietary proteins, which was two‐fold higher than the RDA median (i.e., 0.6 g kg^−1^ day^−1^). In a previous study, we have shown that reducing protein intake from 1.6 to 0.95 g kg^−1^ day^−1^ was necessary to reduce serum IGF‐1 concentrations in individuals practicing severe CR (Fontana *et al*., 2008). We also found that protein restriction, independently of caloric intake, strongly inhibits tumor growth in human xenograft prostate and breast cancer animal models, by lowering serum IGF‐1 and reducing mTOR phosphorylation (Fontana *et al*., 2013). Consistently, it has been shown that in humans the variations of circulating IGF‐1 levels during fasting and refeeding are strongly linked with the rate of urinary urea excretion, a well‐known indicator of protein intake (Clemmons *et al*., 1981).

In this study, we also measured two growth factors, TGF‐β‐1 and PDGF‐AB, implicated in the pathogenesis of cancer (Silver, 1992; Meulmeester & Ten Dijke, 2011), which have previously been shown in observational studies to be reduced by long‐term severe CR in humans (Fontana *et al*., 2004; Meyer *et al*., 2006). Our findings confirmed that CR induces a significant reduction in serum TGF‐β‐1 and PDGF‐AB over time, but the differences between groups were not significant. Data from animal studies have shown that increased serum corticosterone concentration may also play a role in the CR‐induced protective effects against cancer, as adrenalectomy completely reverses the tumor inhibitory effect of CR in some studies (Pashko & Schwartz, 1992, 1996; Stewart *et al*., 2005). It has been hypothesized that a CR‐induced moderate increase in glucocorticoid concentrations may influence cancer development by inhibiting inflammation and promoting the activation of molecular chaperones (e.g., heat‐shock proteins), which ensure proteostasis (Sapolsky *et al*., 2000; Rhen & Cidlowski, 2005). Data from two previous small, short‐term studies have shown that CR‐induced weight loss does not increase cortisol concentration in overweight men and women (Weiss *et al*., 2006; Tam *et al*., 2014). In contrast, this study shows that 1 year of CR slightly but significantly increases serum cortisol level in young and middle‐aged lean or slightly overweight men and women. This ~6% elevation in cortisol, however, was transient because no change in serum cortisol concentration was detected after 2 years of CR.

In conclusion, data from this large randomized clinical trial showed that long‐term CR does not reduce serum IGF‐1 concentration, but causes a reduction in IGF‐1 bioavailability by substantially and persistently increasing IGFBP‐1 levels in young and middle‐aged lean or slightly overweight men and women. These data also showed that long‐term CR results in mild and transient increase in serum cortisol concentrations. More studies are needed to understand the biological implications of these metabolic adaptations on cancer risk, health, and longevity, and whether or not other nutritional interventions (e.g., protein restriction or intermittent fasting) reduce serum IGF1 concentration in humans.

## Experimental procedures

Figure 1 shows the CONSORT diagram which has been reported previously (Ravussin *et al*., 2015). Briefly, CALERIE was an intensive multicenter randomized, controlled trial with the aim of determining the effects of 25% CR (a reduction in energy intake to 25% below the individual's baseline level) over a 2‐year period in healthy volunteers. Men were required to be between 20 and 50 years, and women between 20 and 47 years, of age, with a BMI between 22 and 27.9 kg m^−2^. The study protocol (NCT00427193) was approved by the Institutional Review Boards of Washington University (St. Louis, MO, USA), Tufts University (Boston, MA, USA), Pennington Biomedical Research Center (Baton Rouge, LA, USA), and Duke University (Durham, NC, USA). Study oversight was provided by a Data and Safety Monitoring Board. The participants provided written informed consent.

### Treatment assignment and intervention

After baseline testing, volunteers were randomized to 25% CR or *ad libitum* control groups with a 2:1 allocation in favor of the CR group. Randomization was stratified for site, sex, and BMI. The CR participants were prescribed a 25% reduction in energy intake based on energy requirements determined by doubly labeled water measurements over a 4‐week period. Adherence to the prescribed 25% CR was calculated by the degree to which the study volunteers attained a prescribed weight loss trajectory (average at 1‐year 15.5%, range of 11.9–22.1%) followed by weight maintenance. Furthermore, the accurate level of CR was retrospectively validated by measuring the total daily energy expenditure (TDEE) by doubly labeled water and adjusting TDEE for changes in body composition (Racette *et al*., 2012).

### Body weight and body composition

Body weight was measured fasting in the morning in a light gown. Fat mass (FM) and fat‐free mass (FFM) were measured by DXA using Hologic 4500A, Delphi W or Discovery A scanners (Hologic QDR 4500A; Hologic, Bedford, MA). Scans were analyzed at University of California San Francisco, also responsible for centralized quality control. Machine performance was monitored with baseline and longitudinal phantom cross‐calibrations.

### Dietary intake

Dietary intakes were determined using 6‐day food diaries which were analyzed with Nutrition Data System for Research (Minneapolis, MN, USA).

### Assays

Serum blood samples were obtained after fasting overnight and analyzed in a central laboratory. Commercially available ELISA kits were used to measure IGF‐1, IGF‐binding protein 1, (IGFBP‐1) and IGFBP‐3 (DSL/Beckman Coulter, Brea, CA, USA), and PDGF‐AB and TGF‐β‐1 (R&D Systems, Minneapolis, MN, USA). Cortisol was measured by chemiluminescent immunoassay (ADVIA Centaur, Bayer Health Care, Deerfield, IL).

### Statistical analysis

The same statistical methodologies used in the parent RCT were applied (Racette *et al*., 2012). Briefly, Wilcoxon and the Fisher exact tests were used to evaluate between‐group differences with respect to baseline characteristics. Repeated measures analysis of covariance was applied with change from baseline as the dependent variable, and treatment, time, and the treatment × time interaction as independent variables; site, sex, BMI stratum, and the baseline value were included as covariates. Hypotheses of specific interest, for example, between‐group differences at the individual time points and within‐group changes over time were tested by defining contrasts among the regression parameters; predicted mean change ± standard error are the adjusted values from this model. For any outcome, type‐I error was controlled using a hierarchical gatekeeping strategy (Dmitrienko *et al*., 2011). The treatment × visit interaction term was tested first. If significant, then following standard statistical practice, between‐group differences at each time point were tested at α = 0.05. If not, the treatment main effect was tested next. If significant, then between‐group differences at each time point were tested at α = 0.05. Otherwise a Bonferroni correction was applied at each time point, with the *P*‐values adjusted by multiplying the nominal *P*‐value by the number of tests (truncated at 1.0) (Wright, 1992). Within‐group changes from baseline to the follow‐up visits, however, fell outside this hierarchy and were always protected by a Bonferroni correction.

## Funding source

This study was supported by National Institute on Aging Cooperative Agreements U01‐AG‐020487, U01‐AG‐020478, U01‐AG‐020480, and U01‐AG‐022132 and National Institutes of Health Grants MO1‐RR00036, P30‐DK‐056341 and UL1RR024992.

## Author contributions

The Corresponding Author attests that the authors had access to all the study data, takes responsibility for the accuracy of the analysis, and had authority over manuscript preparation and the decision to submit the manuscript for publication. The content of this article is solely the responsibility of the authors and does not necessarily represent the official views of the National Institute on Aging or the National Institutes of Health.

## Conflict of interest

The authors hereby declare that there was no conflict of interest associated with this study.
